# *KRAS* mutation effects on the 2-[18F]FDG PET uptake of colorectal adenocarcinoma metastases in the liver

**DOI:** 10.1186/s13550-020-00707-0

**Published:** 2020-11-23

**Authors:** M. Popovic, O. Talarico, J. van den Hoff, H. Kunin, Z. Zhang, D. Lafontaine, S. Dogan, J. Leung, E. Kaye, C. Czmielewski, M. E. Mayerhoefer, P. Zanzonico, R. Yaeger, H. Schöder, J. L. Humm, S. B. Solomon, C. T. Sofocleous, A. S. Kirov

**Affiliations:** 1 Memorial Sloan Kettering Cancer Center, 1275 York Avenue, New York, NY 10065 USA; 2 Cornell University, Ithaca, NY 14850 USA; 3 Vassar Brothers Medical Center, Poughkeepsie, NY 12601 USA; 4 Lebedev Physical Institute RAS, Moscow, Russia 119991; 5 Institute of Radiopharmaceutical Cancer Research, Helmholtz-Zentrum Dresden-Rossendorf, 01328 Dresden, Germany; 6 Department of Radiology, Memorial Sloan Kettering Cancer Center, 1275 York Avenue, New York, NY 10065 USA; 7 Department of Epidemiology & Biostatistics, Memorial Sloan Kettering Cancer Center, 1275 York Avenue, New York, NY 10065 USA; 8 Department of Pathology, Memorial Sloan Kettering Cancer Center, 1275 York Avenue, New York, NY 10065 USA; 9 Technology Division, Memorial Sloan Kettering Cancer Center, 1275 York Avenue, New York, NY 10065 USA; 10 Department of Medicine, Memorial Sloan Kettering Cancer Center, 1275 York Avenue, New York, NY 10065 USA

**Keywords:** PET, Colorectal adenocarcinoma, Liver metastases, *KRAS* mutations

## Abstract

**Background:**

Deriving individual tumor genomic characteristics from patient imaging analysis is desirable. We explore the predictive value of 2-[18F]FDG uptake with regard to the *KRAS* mutational status of colorectal adenocarcinoma liver metastases (CLM).

**Methods:**

2-[18F]FDG PET/CT images, surgical pathology and molecular diagnostic reports of 37 patients who underwent PET/CT-guided biopsy of CLM were reviewed under an IRB-approved retrospective research protocol. Sixty CLM in 39 interventional PET scans of the 37 patients were segmented using two different auto-segmentation tools implemented in different commercially available software packages. PET standard uptake values (SUV) were corrected for: (1) partial volume effect (PVE) using cold wall-corrected contrast recovery coefficients derived from phantom spheres with variable diameter and (2) variability of arterial tracer supply and variability of uptake time after injection until start of PET scan derived from the tumor-to-blood standard uptake ratio (SUR) approach. The correlations between the *KRAS* mutational status and the mean, peak and maximum SUV were investigated using Student’s *t* test, Wilcoxon rank sum test with continuity correction, logistic regression and receiver operation characteristic (ROC) analysis. These correlation analyses were also performed for the ratios of the mean, peak and maximum tumor uptake to the mean blood activity concentration at the time of scan: SUR_MEAN_, SUR_PEAK_ and SUR_MAX_, respectively.

**Results:**

Fifteen patients harbored *KRAS* missense mutations (*KRAS*+), while another 3 harbored *KRAS* gene amplification. For 31 lesions, the mutational status was derived from the PET/CT-guided biopsy. The Student’s *t* test* p* values for separating *KRAS* mutant cases decreased after applying PVE correction to all uptake metrics of each lesion and when applying correction for uptake time variability to the SUR metrics. The observed correlations were strongest when both corrections were applied to SUR_MAX_ and when the patients harboring gene amplification were grouped with the wild type: *p* ≤ 0.001; ROC area under the curve = 0.77 and 0.75 for the two different segmentations, respectively, with a mean specificity of 0.69 and sensitivity of 0.85.

**Conclusion:**

The correlations observed after applying the described corrections show potential for assigning probabilities for the *KRAS* missense mutation status in CLM using 2-[18F]FDG PET images.

## Background

The value of functional images for personalized therapy is limited by cancer histological and genomic heterogeneity [1, 2]. Gathering information from medical images regarding proliferation rate, differentiation and heterogeneity, or genomic profile of malignant tumors [3, 4] potentially improves selection of the appropriate treatment pathways for targeted therapies while minimizing the need and risks of interventional procedures and maximizing patient comfort [5].

Colorectal cancer (CRC) patients with Kristen rat sarcoma viral gene (*KRAS*) mutant tumors are associated with lack of response to anti-epidermal growth factor receptor (anti-EGFR) antibody therapy [6–10]. It has been also shown that *KRAS* mutations are a significant predictor of overall survival in metastatic CRC and of recurrence after surgery or radiofrequency ablation of colorectal cancer liver metastases (CLM) [11, 12]. *KRAS* mutation has been associated with relatively higher rates of local failure or positive resection margins after thermal ablation or resection of CLM [11, 12]. Recently, others have found that the coexistence of *KRAS* mutation with increased 2-[18F]FDG uptake is a negative prognostic factor in primary CRC [13]. *KRAS* mutation status has a predictive value also for image-guided ablation for lung adenocarcinoma [14].

**Fig. 1:**
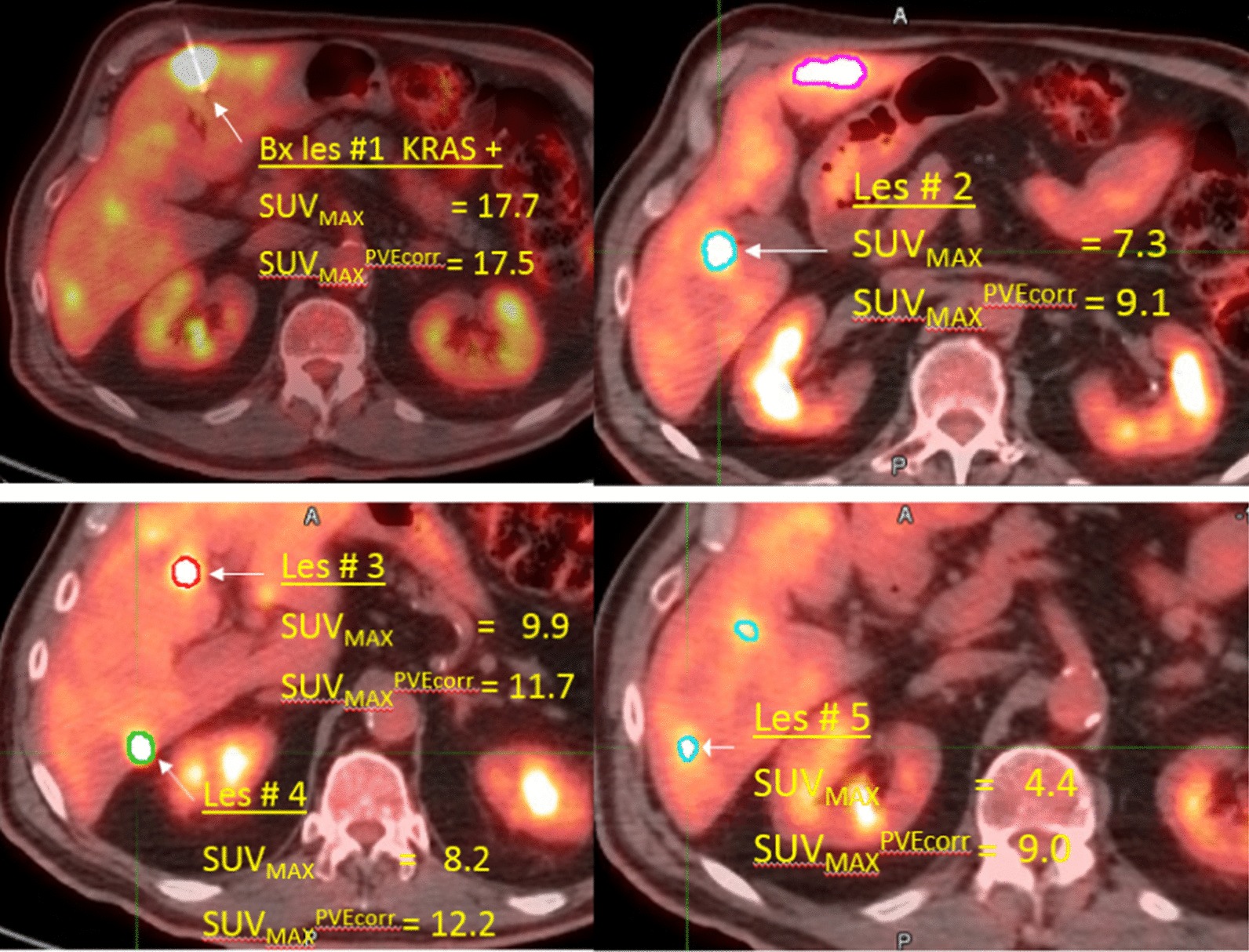
Fused PET/CT images of multiple liver metastases in a colorectal cancer case. Only one of the lesions was biopsied (top left) and showed *KRAS* mutant status. Segmentation contours for four lesions are shown. SUV_MAX_ before and after PVE correction is quoted for each lesion. The PVE correction reduces SUV_MAX_ for the largest lesion due to the positive bias of maximum uptake values compared to the mean (Fig. 3)

While primary and metastatic CRC sites have a high concordance for *KRAS* mutations, meta-analyses suggest the concordance may not be 100%. An initial high (100%) *KRAS* genomic concordance has been observed between the primary tumor and secondary lesions in metastatic CRC [15, 16]. A subsequent meta-analysis showed that the level of concordance may vary, reporting an overall concordance of 94.1% between 986 pairs of primary and distant metastasis from 17 publications [16]. In a more recent review, Mao et al. [17] have observed a pooled concordance rate of 92%. These data suggest that occasionally *KRAS* mutant primary tumors harbored *KRAS* wild-type metastasis and, as such, could potentially benefit from anti-EGFR treatment [17]. Specifically for CLM, these authors found 8.0% false positive (wild-type or normal expression in primary tumor but mutant or loss of expression in metastases) and 9.7% false negative (mutant or loss of expression in primary tumor but wild-type or normal expression in metastases) rates [17]. Additionally, patients treated with targeted therapies, including EGFR, HER2 and BRAF targeting combinations, have been reported to develop *KRAS* mutations at resistance, often in a heterogeneous pattern involving some lesions [18, 19]. Deriving the genomic properties through metabolic imaging of individual lesions may optimize subsequent interventions (Fig. 1).

*KRAS* mutations appear to be related to the glucose metabolism [20]. Therefore, *KRAS* mutant lesions may be distinguishable using images of metabolic activity. The manifestation of *KRAS* mutations in PET images has been studied for non-small cell lung cancer (NSCLC) patients [21, 22] as well as for CRC [13, 23–30]. For NSCLC, one study showed that a multivariate model, including 2-[18F]FDG SUV_MEAN_, may be used as a predictive marker for *KRAS* mutations [21]. However, others found that* EGFR* mutations correlate with NSCLC PET image features, whereas *KRAS* mutations do not [22]. Similarly for CRC, one investigation of the effect of *KRAS* mutations on 2-[18F]FDG uptake of CRC lesions found no correlation [23], while others found statistically significant correlations for primary tumors [13, 24–26]. Several SUV features including SUV_MAX_ and SUV_MEAN_ were found to significantly correlate with the presence of *KRAS* mutations for newly diagnosed rectal cancer [27], although predictive value of the 2-[18F]FDG uptake was low. Other investigations showed statistically significant separation of the *KRAS*-mutated tumors by SUV_MAX_ and SUV_PEAK_ when a C-reactive protein (CRP) upper threshold of 6 mg/L was introduced [28], or when SUV_MAX_ was used in a decision tree also utilizing CT texture and blood flow, derived from dynamic contrast-enhanced CT [29]. For CRC metastases, SUV_MAX_ was significantly associated with *KRAS* mutations when considering only tumors larger than 10 mm in diameter in order to minimize bias due to the partial volume effect (PVE) [30]. Primary tumor SUV_MAX_ was found to be significantly higher in CRC subjects with *KRAS* mutation compared to wild type [13]. None of the above studies were specific to CRC liver metastases (CLM).

We focus on evaluating *KRAS* effects on 2-[18F]FDG uptake specifically for CLM. In addition, we apply corrections to the 2-[18F]FDG uptake for: (1) partial volume effect and (2) time of tracer uptake from injection to PET scan acquisition. The latter is important for the investigated dataset which contains only PET/CT images obtained in the interventional setting during PET/CT-guided ablations or biopsies for which the uptake time can vary. Biopsies and ablations performed under real-time PET/CT guidance are useful to target tumors with poor conspicuity in non-functional/anatomic only imaging [31–35]. If the specimen from a biopsy during such a procedure is subjected to genomic profiling, the molecular and metabolic data are obtained simultaneously for the same lesion, thereby removing uncertainty for the molecular status of the imaged lesion.

## Methods

### Patients

The records of thirty-seven (37) patients who underwent PET/CT-guided biopsies of colorectal adenocarcinoma liver metastases (CLM) in the period between April 2011 and June 2019 with molecular pathology reports were reviewed under an Institutional Review Board-approved retrospective research protocol. Thirty-nine PET/CT scans were analyzed since two patients underwent a second PET/CT-guided biopsy for new liver lesions, which were also included in the analysis. Altogether 60 CLM were segmented in these 39 interventional PET scans using two different PET segmentation tools. Twelve of the included lesions were in the vicinity of previously treated (ablated or resected) region of the liver.

The *KRAS* mutational status for each case was extracted from the molecular pathology report of each case. Thirty-one (31) of the 60 lesions had mutational status assigned from the PET/CT-guided procedure. Other lesions in the same cases as well as cases for which the mutational status was known from another specimen were kept in since the probability for mislabeling is small. The investigated dataset is a compromise between reducing the number of potentially mislabeled cases (from about 10% to about 5%) and having a sufficient number of cases for the analysis.

### PET/CT-guided biopsies

In PET/CT-guided biopsy procedures, after the initial PET/CT scan, the interventional radiologist places the needle by targeting the PET-avid region of the lesion after which a second CT scan with the needle in place is performed and fused with the pre-insertion PET, PET_pre_, for verification of the needle placement [33]. In cases where mis-registration with PET_pre_ is suspected due to motion, a second real-time PET with the needle in place may be obtained [36].

All PET/CT-guided biopsies were performed at the same interventional PET/CT scanner (Discovery 690, GE Healthcare, Waukesha, Wisconsin). The average injected 2-[18F]FDG activity (mean ± SD) was 152.0 ± 52.4 MBq (4.1 ± 1.4 mCi). The post-injection residual activity in the syringe was measured only for one protocol case (6.2 MBq for case # 2), while a residual activity of 11.1 MBq (0.3 mCi) was assumed for all other cases based on our current clinical procedures. Uptake times varied from 40.7 to 205.7 min (mean 83.8 min, std 34 min) due to interventional work specifics: Optimizing the flow of the interventions and patient safety and emergency procedures have a higher priority.

PET scans were centered in the liver and obtained for one or two bed positions consisting of 47 transverse slices, 3.27 mm thick with 11-slice overlap. Transaxial field of view of 70 cm and 128 × 128 image matrix (5.47 × 5.47 mm pixels) were used for all cases. The acquisition time typically varied from 3 to 5 min per bed for the pre-needle insertion PET scans included in this study. PET scans with breath hold [33] were excluded since their number was very small and breath hold affects the SUV. The reconstruction settings were the same for all scans (time-of-flight on, two iterations, 16 subsets, sharpIR system modeling, post-filter: 6.4 mm, axial filter: heavy).

### Molecular pathology

Molecular pathology reports were obtained from the patients’ medical record and reviewed for all 37 patients. If the patients had more than one pathology report, the report from the PET/CT-guided biopsy of the targeted CLM or, if not available, the report closest in time to that biopsy was used. *KRAS* mutation status was determined by clinically validated molecular assays including the mass spectrometry-based Sequenom technologies [37] test only (*n* = 5) and the next-generation targeted exome sequencing by Memorial Sloan Kettering-Integrated Mutation Profiling of Actionable Cancer Targets (MSK-IMPACT) [38] with or without Sequenom (*n* = 32). In addition to *KRAS* missense mutations, MSK-IMPACT also detects *KRAS* copy number alterations such as whole gene amplifications. The *KRAS* mutation status for each case was noted but was not provided to the operators performing tumor segmentation.

### PET image analysis

Sixty CLM were segmented using the PET-edge tool of MIM (MIM Software, Inc., Cleveland, Ohio) and a fixed SUV threshold in Hermes Gold LX (Hermes Medical Solutions, Stockholm, Sweden). Two different researchers performed the segmentations using the two packages and were blinded to the patient mutational status and to each others’ results. All automatically generated contours were visually inspected, and small manual corrections were applied if needed. In Hermes, a threshold of SUV = 4.0 or higher was used: The segmentation thresholds for three higher background cases were increased to avoid unrealistically large volumes.

Mean and peak standard uptake values (SUV, normalized to patient weight) as obtained from the two segmentation methods and the maximum SUVs obtained in Hermes were recorded for all target CLM. When SUV_PEAK_ was not available due to a small CLM volume (37 cases out of the 60 lesions in MIM), SUV_MEAN_ was used as an approximation.

**Fig. 2:**
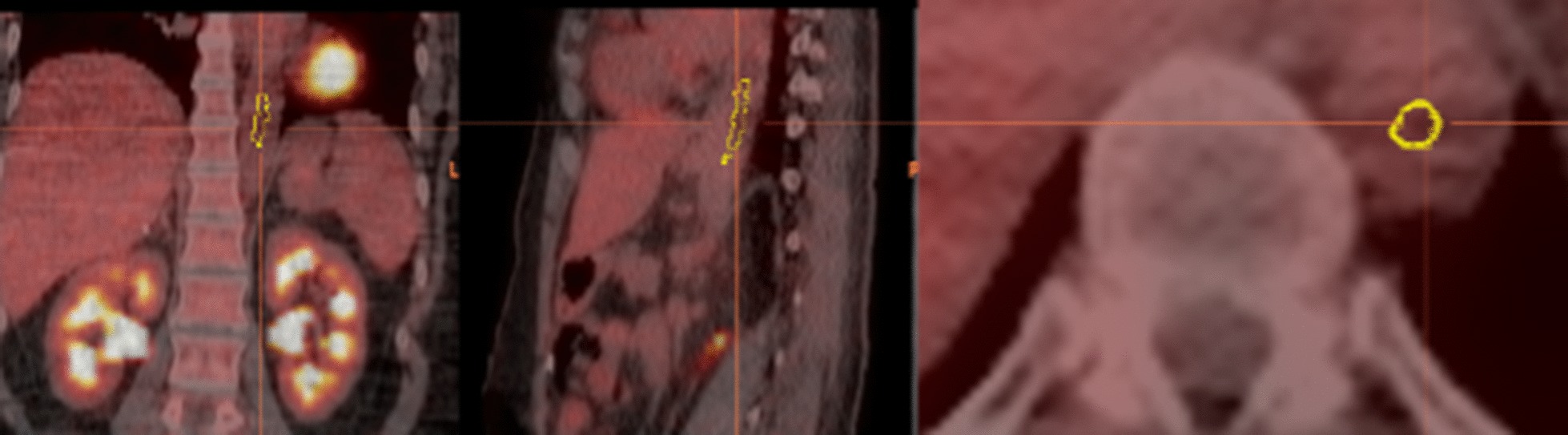
Cropped fused 2-[18F]FDG PET-CT coronal (left), sagittal (middle) and axial (right) images of the descending aorta with contours used for determining the mean blood SUV (case # 15: VOI 2.5 mL, SUV_BLOOD, MEAN_ = 1.9)

The tumor-to-blood SUV ratio approach developed by van den Hoff et al. [39] that corrects for variable uptake time from the FDG injection to the PET scan acquisition, variable tracer supply between subjects and technical uncertainty factors [40] was implemented. To determine the 2-[18F]FDG uptake in the blood, we manually segmented the part of the descending aorta visible in the PET images using Hermes. The contours were drawn at least 5 mm away from the edge of the aorta to minimize PVE effect on SUV mean (Fig. 2).

#### Partial volume effect correction

The limited PET resolution causes a partial volume effect which results in loss of accuracy in recovering the true activity especially in small objects. Since at PET resolution most small liver tumors can be approximated by equal volume spheres, an approximate partial volume effect correction can be applied by using the percent contrast (Q) for different-diameter higher activity spheres placed in uniform background as measured according to the NEMA 2.0 protocol for PET scanner acceptance [41]. During PET acceptance, the measurement is performed at a sphere-to-background activity concentration ratio SBR = 4:1. For our purpose, we filled all spheres of the same phantom with SBR = 2.19:1 (9.284 and 4.240 kBq/mL, respectively) to approximate the mean tumor-to-normal liver ratio in the 39 patient scans. The lung insert in the center of the phantom was also in place with no activity. The phantom was scanned on the same PET scanner where all patients were scanned with two bed positions for 15 min each to reduce image noise. Spherical VOIs with diameters matching those of the inner diameter of the phantom spheres (10, 13, 17, 22, 28 and 37 mm) were centered in the CT images of the phantom spheres and then copied to the same location in the registered PET images using the Hermes software. Then, the mean, peak and maximum SUV for each sphere were recorded to calculate the respective recovery coefficients as described below.

The equation for contrast recovery in NEMA 2-2018 [41] is

1 $${\text{RC}}_{{{\text{MEAN}}}}^{H,j} = \frac{{\frac{{C_{{{\text{MEAN}}}}^{H,j} }}{{C_{B,j} }} - 1}}{{\frac{{a_{H,j} }}{{a_{B,j} }} - 1}}$$

where $C_{{{\text{MEAN}}}}^{H,j}$ and $c_{B,j}$ are the average counts and $a_{H,j}$ and $a_{B,j}$ are the activity concentrations in hot sphere *j* and in the background, respectively, which were rewritten in terms of SUV by introducing the respective constants. Then, acknowledging that for the mean background SUV the recovery coefficient is RC = 1.0, for the partial volume corrected mean SUV for sphere *j*, ${\text{SUV}}_{{{\text{MEAN}}}}^{{{\text{PVEC}},j}}$, we obtain:

2 $${\text{SUV}}_{{{\text{MEAN}}}}^{{{\text{PVEC}},j}} = \frac{{{\text{SUV}}_{{{\text{MEAN}}}}^{H,j} }}{{{\text{RC}}_{{{\text{MEAN}}}}^{H,j} }} - {\text{SUV}}_{{{\text{MEAN}}}}^{B,j} \left( {1/{\text{RC}}_{{{\text{MEAN}}}}^{H,j} - 1} \right),$$

where ${\text{SUV}}_{{{\text{MEAN}}}}^{H,j}$ and ${\text{SUV}}_{{{\text{MEAN}}}}^{B,j}$ are the measured mean SUVs for hot sphere *j* and the background around it.

In analogy to (1), recovery coefficients can be defined also for the maximum and for the peak measured activities:

3 $${\text{RC}}_{{{\text{MAX}}\,{\text{or}}\,{\text{PEAK}}}}^{H,j} = \frac{{\frac{{C_{{{\text{MAX}}\,{\text{or}}\,{\text{PEAK}}}}^{H,j} }}{{C_{B,j} }} - 1}}{{\frac{{a_{H,j} }}{{a_{B,j} }} - 1}},$$

which will allow to apply partial volume correction to SUV_PEAK_ and SUV_MAX_ using:

4 $${\text{SUV}}_{{{\text{MAX}}\,{\text{or}}\,{\text{PEAK}}}}^{{{\text{PVEC}},j}} = \frac{{{\text{SUV}}_{{{\text{MAX}}\,{\text{or}}\,{\text{PEAK}}}}^{H,j} }}{{{\text{RC}}_{{{\text{MAX}}\,{\text{or}}\,{\text{PEAK}}}}^{H,j} }} - {\text{SUV}}_{{{\text{MEAN}}}}^{B,j} \left( {1/{\text{RC}}_{{{\text{MAX}}\,{\text{or}}\,{\text{PEAK}}}}^{H,j} - 1} \right)$$

**Fig. 3:**
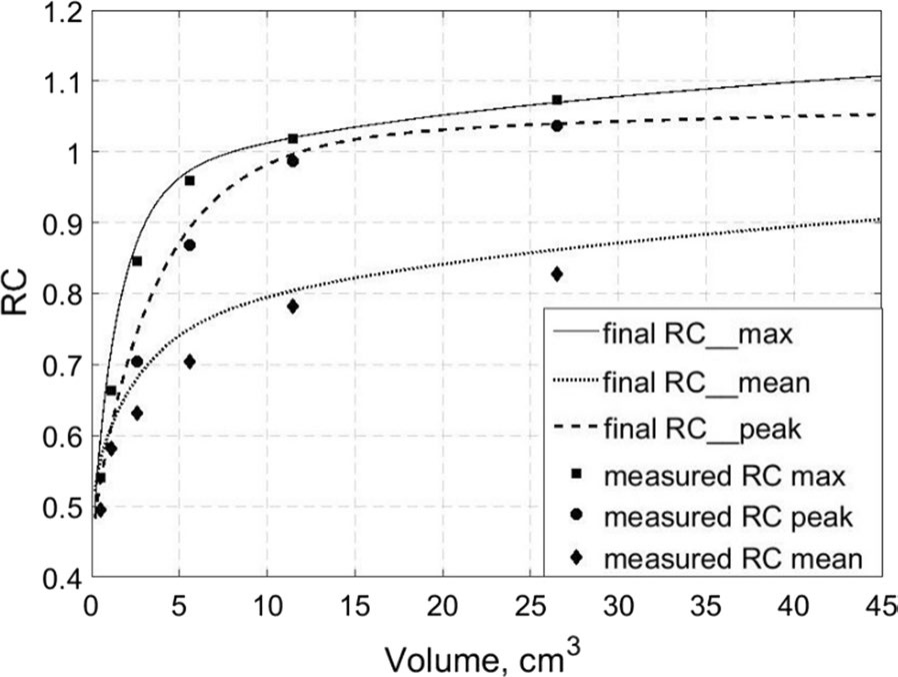
Recovery coefficients (RC) used for applying PVE correction to the tracer uptake of each lesion. The symbols represent the measured RC for each NEMA NU-2 image quality phantom sphere. The final recovery coefficients used for PVE correction (lines) are obtained by applying a correction for the cold walls of the phantom spheres obtained by using a RC model [42] to compute the recovery coefficient ratio for spheres with and without walls

The measured recovery coefficients for SUV_MEAN_, SUV_PEAK_ and SUV_MAX_ (Eqs. 1 and 3) are plotted for each sphere in Fig. 3. Corrections to these recovery coefficients were applied for the 1 mm thickness of the cold sphere walls using a RC model obtained by convolution of the resolution PSF with the spheres given the known sphere-to-background contrast (lines in Fig. 3) [42]. The cold wall correction was obtained from the ratio of the simulated recovery coefficients for the max, peak and mean SUVs for spheres with the same inner diameters with and without 1 mm walls, respectively. The sphere external diameters were confirmed by caliper measurements, and the wall thickness was verified using a micro-CT scan for some of the spheres. In the simulations, we performed convolution of a symmetrical Gaussian point-spread function with each sphere and used a sphere-to-background ratio of 2.26, which is close to the midpoint between the ratios in the NEMA phantom and the patients. The cold wall corrections to the RC ranged up to ~ 11.1% and 12.8% for 1 mL lesions for SUV_MEAN_ and SUV_MAX_ and were less than 5% and 0.2% for volumes larger than 10 mL, respectively. For extending the PVE corrections to the few lesions with volumes larger than the 37-mm-diameter sphere (26.52 mL), we followed the RC trends provided by the convolution-based model described above [42]. Note that according to our definition (Eq. 3), the RC for SUV_MAX_ and SUV_PEAK_ are larger than 1.0 for large lesions due to statistical effects (positive bias of maximum relative to mean).

The approximate SUV_MEAN_, SUV_PEAK_ and SUV_MAX_ PVE correction for each lesion with volume *V*_*les*_, *RC*_*eff*_* (V*_*les*_*)* was obtained by interpolation of the final RC curves obtained after cold wall correction (lines in Fig. 3). The mean background SUV for each lesion, ${\text{SUV}}_{{B,{\text{lesion}}}}$, was measured in two PET slices using doughnut-shaped ROIs manually drawn to avoid the visible spill out from the respective lesion. The PVE-corrected SUV_MEAN_, SUV_PEAK_ and SUV_MAX_ for each lesion are then obtained using ${\text{SUV}}_{{B,{\text{lesion}}}}$ and Eqs. (2) and (4). For lesions close to the periphery of the liver, $SUV_{B,lesion}$ was less than the mean normal liver SUV, ${\text{SUV}}_{{B,{\text{mean liver}}}}$. The latter, ${\text{SUV}}_{{B,{\text{mean liver}}}}$, was obtained from large manually drawn ROIs in normal liver far from the lesions and was used for calculating the tumor-to-liver ratio, SUVTLR = SUV_PVEC, lesion_/SUV_*B*, mean liver_, for each lesion.

#### Uptake time correction

We employed the uptake time correction method developed by van den Hoff et al. [39] for CRC liver metastases and validated also for other lesions. For calculating the tumor-to-blood standard uptake ratio (SUR) employed by this method, we derived the blood SUV from the 2-[18F]FDG activity in the descending aorta. Since for liver interventions the PET scan is limited to two bed positions (83 slices) providing an axial field of view (FOV) from the inferior to the superior border of the liver, and we extracted SUV_blood mean_ from an aorta volume at least 0.5 cm away from the aorta surface to minimize PVE, the aorta ROI volumes were smaller (average analyzed volume 1.8 mL) than those previously used [39]. The average SUV values for the descending aorta were 2.2 + − 0.6 mL/g. The injection and scan times were automatically extracted from the image headers using an in-house developed tool (DBbrowser). SURs were calculated as SUR_MEAN_ = SUV_lesion mean_/SUV_blood mean_, SUR_PEAK_ = SUV_lesion peak_/SUV_blood mean_ and SUR_MAX_ = SUV_lesion max_/SUV_blood mean_, using both MIM and Hermes segmentation contours of the lesions and Hermes contours for SUV_blood mean_. A correction for the difference between actual uptake time post-injection, *T*, and standard uptake time $T_{0}$ = 60 min was applied to each lesion’s SUR_MEAN_, SUR_PEAK_ and SUR_MAX_ using the approximations to equations (8) and (10) proposed by van der Hoff et al. [39]: ${\text{SUR}}_{0} = \frac{{T_{0} }}{T}\left( {{\text{SUR}}_{T} - V_{r} } \right) + V_{r} \approx {\text{SUR}}_{T} \frac{{T_{0} }}{T}$ and ${\text{SUV}}_{0} = {\text{SUV}}_{T} \frac{{{\text{SUR}}_{0} }}{{{\text{SUR}}_{T} }}\left( {\frac{{T_{0} }}{T}} \right)^{ - b} \approx {\text{SUV}}_{T} \left( {\frac{{T_{0} }}{T}} \right)^{1 - b}$ with apparent volume of distribution, *V*_r_ = 0.53, and parameter *b* = 0.313 as determined previously [39]. By reducing variance, this approach has led to improved correlations in several studies with similar and larger patient cohorts for different cancer types [43–47]. In one of these studies, which used 90 cases with dual time point 2-[18F]FDG PET, the uptake time varied from 56.4 to 197 min [44].

The lesion volume and the SUV mean, peak and max metrics were extracted immediately after segmentation. Then PVE correction was applied to these values according to the lesion volume. SUVTLR and SUR were calculated using the PVE-corrected mean, peak and max SUV, which was then followed by applying the uptake time correction prior to investigating the correlations.

## Results

### Patient demographics and *KRAS* mutations

The patient demographics are given in Table 1. *KRAS* mutations were observed in 15 (37.8%) and copy number variations in 3 (8.1%) of the 37 patients, respectively (Table 2). In 16 of the 18 patients with *KRAS* mutant tumors (except #9 and #23 in Table 2) and 15 of the 19 patients with *KRAS* wild-type tumors, sequencing was performed on the target CLM from the same PET/CT-guided biopsy. Of the 15 missense mutations, eight are in codon G12, four are in codon G13D, two in codon Q61H and one in codon A146T.

**Table 1:** Patient demographics

Number of patients	37
Number of PET/CT images	39
Age (years)	56.3 ± 11.9
Gender	
Male	27 (73%)
Female	10 (27%)
Weight (kg)	90.3 ± 17.1
Ablation with biopsy	27
Biopsy only	12

**Table 2:** *KRAS* mutations and copy number variations observed in 18 of the 37 patients

Case #	*KRAS* gene alteration and location
2	G13D (c.38G > A) exon 2
5	G12D (c.35G > A) exon 2
6	A146T (c.436G > A) exon 4
7	G12A (c.35G > C) exon 2
8	G12D (c.35G > A) exon 2
9	Whole gene amplification 12p12.1, FC:2.1
11	Q61H (c. 183A > C) exon 3
14	G13D (c.38G > A) exon 2
15	G12V (c.35G > T) exon 2
16	G12D (c.35G > A) exon 2
23	G12D (c.35G > A) exon 2
24	G13D (c.38G > A) exon 2
25	G12D (c.35G > A) exon 2
28	Whole gene amplification 12p12.1, FC: 23.0 (Inv. Panel)
31	Q61H (c.183A > T) exon 3
32	G13D (c.38G > A) exon 2
35	G12C (c.34 G > T) exon 2
37	Whole gene amplification 12p12.1, FC: 8.5

FC, fold change; Inv. Panel, investigational panel

In addition to patient #11(Table 2), two more patients have developed a *KRAS* mutation in codon Q61H after initial testing. Case #1 had no *KRAS* mutation in the primary and later, at the time of CLM targeting PET/CT-guided biopsy, but showed a Q61H exon 3 *KRAS* mutation in a different lesion in a remote section of the liver 2.5 years later during preparation for radioembolization. Similarly, case #31 had no *KRAS* mutation in an older sigmoid rectum specimen (primary) but exhibited also a codon Q61H exon 3 *KRAS* mutation in the targeted CLM at time of the PET/CT-guided biopsy 5 years later. Altogether, both primary tumor and a metastasis were genotyped in 15 cases and the *KRAS* status was concordant in 13/15 (87%) cases.

### PET image analysis

The average tumor volumes obtained with the PET-edge algorithm in MIM and with the SUV threshold algorithm in Hermes are 5.3 mL (min. 0.3, max. 31.3, std. 6.9 mL) and 6.1 mL (min. 0.2, max. 43.0, std. 8.5 mL), respectively. The average lesion diameter from the two segmentations is 22 mm. Before PVE correction, the mean lesion SUV of all 60 lesions was 6.2 ± 2.2 (MIM) and 6.0 ± 1.6 (Hermes) and the mean normal liver SUV is 2.7 ± 0.6 resulting in mean tumor-to-liver ratio of 2.2 and 2.3 for Hermes and MIM lesion contours, respectively. The mean background liver SUV measured in the vicinity of the lesions, ${\text{SUV}}_{{B,{\text{lesion}}}}$, and used for the RC calculation [Eqs. (2) and (4)] is 2.4 ± 0.7. The average SUV values obtained for the descending aorta are 2.2 ± 0.6. The values for the different uptake metrics corresponding to the two segmentation methods for each mutation status are given in Table 3.

**Table 3:** Average 2-[18F]FDG SUV metrics (± 1 STD) for *KRAS* wild-type and missense mutated CLM obtained with two independent volume segmentations (MIM and HERMES)

	Mean uptake		Peak uptake		Max uptake	
	*KRAS* wild-type	*KRAS*+	*KRAS* wild-type	*KRAS*+	*KRAS* wild-type	*KRAS*+
SUV (MIM)	5.70 ± 1.82	6.91 ± 2.46	6.56 ± 2.61	8.29 ± 4.04	8.63 ± 3.74	11.79 ± 5.17
SUV (HERMES)	5.63 ± 1.46	6.65 ± 1.68	7.09 ± 2.97	9.21 ± 4.33	8.63 ± 3.74	11.79 ± 5.17
SUV MIM + PVEC	7.13 ± 2.62	8.94 ± 3.26	7.63 ± 2.92	9.79 ± 3.93	9.44 ± 3.89	13.09 ± 4.96
SUV (HERMES) + PVEC	7.00 ± 2.12	8.37 ± 1.86	8.18 ± 3.17	10.53 ± 3.95	9.33 ± 3.66	12.63 ± 4.68
SUV (MIM) + PVEC + Time corr	5.98 ± 2.39	7.74 ± 3.18	6.42 ± 2.74	8.44 ± 3.66	7.92 ± 3.57	11.19 ± 4.46
SUV (HERMES) + PVEC + Time corr	5.87 ± 2.14	7.22 ± 1.92	6.88 ± 3.04	8.99 ± 3.44	7.83 ± 3.45	10.77 ± 4.05
SUVTLR (MIM) + PVEC	2.64 ± 1.11	3.54 ± 1.53	2.81 ± 1.20	3.92 ± 1.91	3.51 ± 1.66	5.28 ± 2.61
SUVTLR (HERMES) + PVEC	2.62 ± 1.08	3.35 ± 1.16	3.06 ± 1.43	4.27 ± 2.16	3.49 ± 1.63	5.12 ± 2.56
SUR (MIM) Time corr	2.10 ± 0.89	2.77 ± 0.83	2.43 ± 1.24	3.30 ± 1.43	3.20 ± 1.74	4.66 ± 1.81
SUR (HERMES) Time corr	2.06 ± 0.81	2.70 ± 0.67	2.63 ± 1.40	3.65 ± 1.53	3.20 ± 1.74	4.66 ± 1.81
SUR (MIM) + PVEC + Time corr	2.60 ± 1.17	3.59 ± 1.15	2.80 ± 1.35	3.93 ± 1.43	3.47 ± 1.79	5.23 ± 1.79
SUR (HERMES) + PVEC + Time corr	2.55 ± 1.07	3.42 ± 0.85	3.01 ± 1.52	4.23 ± 1.46	3.43 ± 1.75	5.06 ± 1.72

*KRAS* gene amplification cases (*n* = 3) are grouped with wild type. Average metrics values after PVE and uptake time correction are provided for the derivative metrics tumor-to-liver ratio (SUVTLR) and tumor-to-blood ratio (SUR) as well

### Correlations

Since only *KRAS* missense mutations were reliably shown to affect the outcome of anti-EGFR therapy, below we present the results when *KRAS* gene amplification cases are grouped with wild type. The results when the three cases carrying *KRAS* gene amplification and no KRAS missense mutations are grouped with *KRAS* missense mutations are presented in the supplement.

The correlations observed between the investigated SUV metrics and the *KRAS* mutational status are presented in Table 4 using Student’s *t* test and Wilcoxon rank sum test with continuity correction *p* values and area under the curve (AUC) of the receiver operating characteristic (ROC). The metrics presented in Table 4 are ordered with decreasing *p* values from top to bottom. This correlates with the increase in the AUC values which are presented only for the bottom row and the rightmost column.

**Table 4:** Statistics for the separation of *KRAS* missense mutations based on 2-[18F]FDG uptake when *KRAS* gene amplification is grouped with wild type for all 60 lesions in 39 interventional PET/CT scans

Parameter	Mean		Peak		Max		Statistic
	MIM	HERMES	MIM	HERMES	MIM	HERMES	
SUV No corr	0.05	0.02	0.07	0.04	0.01	0.01 0.01 0.692	Stud. *t* test Wilc. R. Sum AUC
SUV PVE corr	0.03	0.01	0.03	0.02	0.004	0.006 0.005 0.715	Stud. *t* test Wilc. R. Sum AUC
SUVTLR PVE corr	0.02	0.02	0.02	0.02	0.006	0.009 0.004 0.722	Stud. *t* test Wilc. R. Sum AUC
SUV PVE + time corr	0.03	0.01	0.03	0.02	0.005	0.006 0.005 0.714	Stud. *t* test Wilc. R. Sum AUC
SUR time corr	0.004	0.002	0.02	0.01	0.003	0.003 0.002 0.733	Stud. *t* test Wilc. R. Sum AUC
SUR PVE + time corr	0.002 0.003 0.725	0.0009 0.0004 0.770	0.004 0.004 0.723	0.003 0.002 0.738	0.0005 0.0005 0.766	0.0008 0.001 0.753	Stud. *t* test Wilc. R. Sum AUC

Student’s *t* test *p* values are given for all metrics, while Wilcoxon rank sum test with continuity correction *p* values (abbreviated to Wilc. R. Sum) and AUC are given as a second and third line number only for PVE- and time-corrected SUR (bottom row) and for the maximum values derived from the Hermes segmentation contours. Values obtained after PVE and uptake time correction as well as for derivative metrics as tumor-to-liver ratio (SUVTLR) and tumor-to-blood ratio (SUR) are provided

**Fig. 4:**
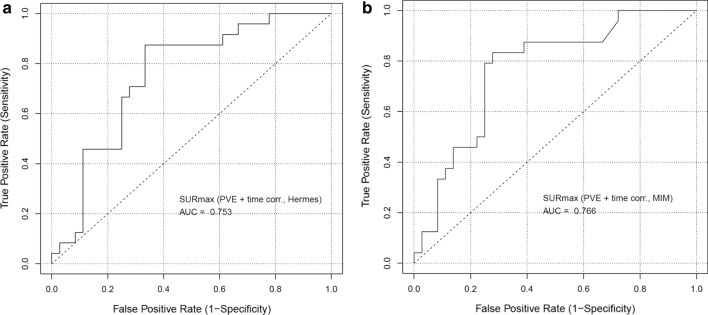
Receiver operating characteristic (ROC) curves and AUC values for predicting CLM *KRAS* missense mutations (*KRAS* gene amplification is grouped with wild type) based on all 60 lesions and SUR_MAX_ with both corrections for Hermes (**a**, left) and MIM segmentations (**b**, right)

The mean coefficient of variation of the AUC between the two segmentation methods for SUR_MEAN_, SUR_PEAK_ and SUR_MAX_ with both PVE and uptake time corrections is 4.3, 1.5 and 1.2% (from the AUC values for SUR_PVE+time corr_ in Table 4), respectively. ROC curves and AUC for SUR_MAX_ for *KRAS* mutation prediction are shown in Fig. 4. The sensitivities and specificities for predicting *KRAS* mutations by the SUR-derived metrics are shown in Table 5.

**Table 5:** Specificities and sensitivities for predicting *KRAS* missense mutations based on all 60 lesions using SUR metrics after PVE and uptake time corrections

	Specificity	Sensitivity	Sensitivity + Specificity
SUR_MEAN_			
MIM	0.72	0.79	1.51
Hermes	0.81	0.71	1.51
SUR_PEAK_			
MIM	0.67	0.83	1.50
Hermes	0.67	0.88	1.54
SUR_MAX_			
MIM	0.72	0.83	1.56
Hermes	0.67	0.88	1.54

The rounded values obtained for contours drawn by different operators using different types of segmentation algorithms within different software platforms are listed

**Fig. 5:**
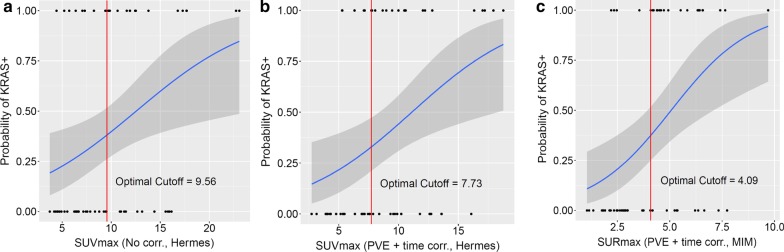
Logistic regression curves based on all 60 lesions for SUV_MAX_ without any corrections (**a**), with both PVE and uptake time corrections (**b**), and for SUR_MAX_ with both corrections (**c**), when *KRAS* gene amplification is grouped with wild-type mutations. Dark gray shaded areas represent the 95% confidence intervals around the probability values. Individual data points are shown with dots at probability levels of 0.00 and 1.00

Logistic regression curves for SUV_MAX_ with and without PVE and uptake time corrections and for SUR_MAX_ with both corrections are shown in Fig. 5. We see some dependence of the logistics regression optimal cutoff values, on the segmentation methods used. The optimal cutoff values for SUR after PVE and time correction for MIM versus Hermes segmentation are: 3.04 versus 3.11 for SUR_MEAN_, 2.89 versus 3.11 for SUR_PEAK_ and 4.09 versus 3.78 for SUR_MAX._ The optimal cutoff however did not change between the two types of grouping for the *KRAS* gene amplification cases: with wild type as presented in Fig. 5 and with KRAS+ as presented in Additional file 1: Fig. S2.

In 31/39 interventional PET/CT scans analyzed, the mutational status of the targeted lesion was determined from the specimen extracted from the same procedure. Applying the analysis for SUV_MAX_ only to the 31 lesions with accurately known *KRAS* mutational status also showed separation of the two groups when two remaining amplification cases are grouped with the wild type (average Student’s *t* test *p* values between the two segmentations: 0.003 for SUV_MAX_ with no corrections, 0.020 after both corrections and 0.011 for SUR_MAX_ after both corrections).

If we remove the 12 lesions in the vicinity of previously treated parts of the liver and analyze the remaining 48 lesions, the Student’s t test *p* values for separating *KRAS*+ mutations from wild type and whole gene amplification based on maximum uptake are: 0.015 for SUV, 0.006 for SUV_PVE_, 0.008 for SUVTLR, 0.005 for SUV_PVE+time cor._, 0.005 for SUR_time cor._ and 0.001 for SUR_PVE+time cor._ In this case, the mean AUCs between the two segmentations are 0.74 for SUV_MAX, PVE + Time corr_ and 0.77 for SUR_MAX,PVE+time cor_. If within this group of 48 lesions *KRAS* whole gene amplification cases are grouped with *KRAS*+, statistical significance is lost.

When the three *KRAS* gene amplification cases are grouped with *KRAS* missense mutations, the *p* values are higher and the specificity and sensitivity and the AUC values are lower (see Additional file 1: Tables S1, S2 and Fig. S1).

## Discussion

For the investigated patient cohort, applying PVE corrections to the SUV leads to better separation of the *KRAS* mutations for all three SUV- and SUR-based metrics: mean, peak and max (Tables 3 and 4, Figs. 4 and 5). Applying uptake time correction leads to noticeably better separation of the two groups only for the SUR metrics but not for the SUV metrics (Table 4). PVE-corrected tumor-to-liver ratios, SUVTLR, performed similarly to PVE-corrected SUV. Using the uptake time-corrected tumor-to-blood ratio, SUR, both with and without PVEC result in better separation of the two groups than PVE- and uptake time-corrected SUV. This indicates that uptake time variability is not the only relevant factor affecting the correlation between the chosen uptake metric and the tumor’s metabolic rate. The removal of inaccuracies related to injected activity due to the residual activity assumption, scanner calibration and body mass/weight as well as accounting for inter-subject variability of the arterial tracer supply as achieved by the use of the SUR metric is of key importance as well [40]. In fact, the logistic regression analysis (Fig. 5) shows that for the current patient cohort only the PVE- and uptake time-corrected SUR_MAX_ provides sufficient separation of the two groups to make mutation predictions from the 2-[18F]FDG uptake in PET images.

Using SUR_MAX_ and applying the corrections described above seem to have allowed for better separation of *KRAS* mutated from wild-type CRC liver lesions than that demonstrated previously [24–27, 30] (Table 4). When *KRAS* gene amplification is grouped with wild type, the PVE- and uptake time-corrected SUR_MAX_ gives the highest predictive value: Between the two segmentations, the mean specificity is 0.694 and the mean sensitivity is 0.854 (sum 1.548, Table 5). For SUR_MEAN_ and SUR_PEAK_, the mean specificity + sensitivity is slightly lower: 1.51 and 1.52.

Another factor we consider helpful in achieving better separation of the *KRAS*-mutated cases is an increased accuracy in labeling the mutational status. In 31/39 interventional PET/CT scans analyzed, the mutational status of the targeted lesion was determined from the specimen extracted from the same procedure. According to previous publications, about 6–10% of the remaining 29 lesions may have mutational status different from that determined in other lesions of the same patient [17]. Assuming that the overall discordance rate is approximately 10% [17], and since the mutational status of 31/60 lesions is precisely known, only 5% (0.1 *(60–31) = 2.9) of the 60 lesions may be potentially mislabeled. This decreases in half the general discordance rate. Thus, including target tumors with a precisely known mutational status (52% in this cohort) increases the accuracy of the dataset and may have contributed to better separation of the two mutations.

In our dataset, the *KRAS* missense mutations seem better separated (lower *p* values, higher AUC in Table 4 and Fig. 4) when the cases with *KRAS* gene amplification (3 of the 32 cases tested by MSK-IMPACT) are grouped with the wild type. This is understandable since *KRAS*-mutated cells were shown to exhibit enhanced glucose uptake [20] and these missense mutations were also found to be mutually exclusive with *KRAS* whole gene amplification in this as well as in other patient cohorts [48]. The optimal cutoff values in the logistic regression analysis were not affected by reassigning the *KRAS* gene amplification cases to the *KRAS* mutant group (Fig. 5 and Additional file 1: Fig. S2) probably due to the small number of gene amplification cases. The mean specificity and sensitivity between the two segmentations for PVE- and time-corrected SUR_MAX_ in this case are 0.69 and 0.77, respectively (Additional file 1: Table S2), compared to 0.69 and 0.85 for grouping with the wild type (Table 5).

One study has shown association of *KRAS* gene amplification with lack of response to anti-EGFR therapy in all four gene amplification cases of 53 non-responding cases; however, according to the authors this was not statistically significant and for these cases it was mutually exclusive with *KRAS* missense mutations [48]. *KRAS* gene amplification was seen in 0.7% of cases with “*de novo* resistance to anti-EGFR treatment” [49]. However, while *KRAS* amplification is a recurrent event in CRC and designated as likely oncogenic by the OncoKB knowledge base [50], at present, *KRAS* amplification per se is not a contraindication for anti-EGFR therapy [51] and its clinical significance remains uncertain.

In the investigated cohort, most of the observed *KRAS* missense mutations are in codon 12 (*n* = 8), but codons 13 (*n* = 4), 61 (*n* = 2) and 146 (*n* = 1) were also represented. While for mutations in codon 61 the reduction in response rate to cetuximab plus chemotherapy compared to wild type was found to be significant, that for mutations in codon 146 was not significant for a small number of cases [9]. Also, *KRAS* G13D mutations were shown to have potential positive effect on survival compared to other *KRAS* mutations in a retrospective study; however; further scrutiny of this effect is needed [52, 53]. Therefore, if the above findings of the effect of *KRAS* missense mutation location are confirmed, investigations of the effect of mutation location on 2-[18F]FDG uptake with larger patient cohorts harboring such mutations would be justified.

The ROC and logistic regression curves (Figs. 4 and 5) show that some of the 2-[18F]FDG uptake-based metrics presented have potential to predict *KRAS* missense mutations in CLM. From the investigated metrics, PVE- and uptake time-corrected SUR_MAX_ results in the highest AUC, specificity and sensitivity and therefore seems most promising for mutational status characterization. Making such a prediction based on previous or on intraprocedural PET images can prompt the interventional radiologist to biopsy lesions which may harbor mutations potentially different from those previously established or among different lesions. In this way, early predictions of CLM *KRAS* mutations, especially for cases with multiple lesions, would allow optimization of immediate interventions [11, 14] as well as of future treatments [6–10].

To further establish 2-[18F]FDG uptake as a biomarker of *KRAS*+ status in CLM, it is important to address the limitations of the presented investigation. Several steps in that direction can be taken. One would be to reduce the uncertainty in SUV by requiring residual activity measurement for each patient injection. Another would be including more lesions with specifically known mutational status to further reduce the labeling error and improve statistics. Use of SUR eliminates uncertainties related to injected activity and other technical and patient-related factors. While several studies have shown that using the SUR-based uptake time correction leads to finding better defined correlations by reducing variance in uptake, this work extends the applicability of this approach to larger uptake time differences.

## Conclusion

Reducing the number of potentially mislabeled cases by focusing predominantly on lesions with genomic profile obtained from 2-[18F]FDG PET/CT-guided biopsies, applying partial volume effect and uptake time corrections, and using the ratio of maximum tumor to mean blood uptake in PET/CT scans, allowed for a highly statistically significant separation of colorectal adenocarcinoma liver lesions expressing *KRAS* missense mutations. Future testing with a larger patient cohort and implementation of these algorithms in the clinic may allow to assign probabilities for the *KRAS* mutation status of such lesions from the PET images. This may lead to further optimization of interventions and therapy for metastatic colorectal adenocarcinoma in the liver.

## Supplementary information

**Additional file 1:** Results when the* KRAS* gene amplification cases are grouped with* KRAS* missense mutation cases.

## Data Availability

The datasets generated and/or analyzed during the current study are available from the corresponding author on reasonable request.

## Abbreviations

AUC: Area under the curve
*BRAF*: B-Raf
CRC: Colorectal cancer
CLM: Colorectal adenocarcinoma liver metastases
*EGFR*: Epidermal growth factor receptor
HER2: Human epidermal growth factor receptor-2
FOV: Field of view
2-[^18^F]FDG: 2-Deoxy-2-[18F]fluoro-D-glucose
*KRAS*: Kristen rat sarcoma viral gene
*KRAS*+: *KRAS* missense mutation
NSCLC: Non-small cell lung cancer
PET: Positron emission tomography
PSF: Point-spread function
PVE: Partial volume effect
RC: Recovery coefficient
ROC: Receiver operating characteristic
SBR: Sphere-to-background activity ratio
SUV: Standard uptake values
SUR: Tumor-to-blood standard uptake ratios
SUR_MEAN_, SUR_PEAK_, SUR_MAX_: Ratios of the mean, peak and maximum tumor uptake to the mean blood uptake
SUVTLR: Tumor-to-liver uptake ratio
VOI: Volume of interest
