# Supplementary material for: KRAS mutation effects on the 2-[18F]FDG PET uptake of colorectal adenocarcinoma metastases in the liver
Source: EJNMMI Res. 2020 Nov 23;10:142. doi: 10.1186/s13550-020-00707-0 (PMC7683631; doi:10.1186/s13550-020-00707-0)
Supplement: Supplementary file 1 — Additional file 1: Results when the KRAS gene amplification cases are grouped with KRAS missense mutation cases. [file 13550_2020_707_MOESM1_ESM.pdf]

SUPPLEMENT

This supplement contains extensions for the case when *KRAS* gene amplification is grouped with *KRAS* missense mutation. The supplemental information in Tables S1.A and S1.B below can be considered as an extension to Table 4 in the paper. Similarly, Table S.2 is an extension to Table 5, Fig. S1 to Fig. 4 and Fig. S2 to Fig. 5. "MIM" and "Hermes" denote the tool used for segmenting the lesion in the PET images as explained in the paper.

Tables S1A and S1B. Wilcoxon rank sum test with continuity correction p-values (abbreviated to Wilc. R. Sum) and AUC for all 60 lesions in 39 interventional PET/CT scans when *KRAS* gene amplification is grouped with *KRAS* missense mutation. Values obtained without and with PVE and uptake time corrections as well as for derivative metrics as tumor-to-liver ratio (SUVTLR) and tumor-to-blood ratio (SUR) are also provided.

Table S1. A

Parameter	Max HERMES	Statistic
**SUV**<br>**No corrections**	***0.13***<br>***0.614***	***Wilc. R. Sum***<br>***AUC***
**SUV**<br>**PVE corrected**	***0.07***<br>***0.639***	***Wilc. R. Sum***<br>***AUC***
**SUVTLR**<br>**PVE corrected**	***0.08***<br>***0.632***	***Wilc. R. Sum***<br>***AUC***
**SUV**<br>**PVE + time corrected**	***0.07***<br>***0.636***	***Wilc. R. Sum***<br>***AUC***
**SUR**<br>**Time corrected**	***0.04***<br>***0.652***	***Wilc. R. Sum***<br>***AUC***

Table S1. B

	**Mean**		**Peak**		**Max**		
	**MIM**	**HERMES**	**MIM**	**HERMES**	**MIM**	**HERMES**	**Statistic**
SUR	***0.03***	***0.006***	***0.03***	***0.02***	***0.01***	***0.02***	***Wilc. R. Sum***
PVE + time corr.	***0.669***	***0.708***	***0.665***	***0.680***	***0.690***	***0.672***	***AUC***

Table S2. Specificities and sensitivities for predicting *KRAS* mutations based on all 60 lesions using SUR metrics after PVE and uptake time corrections when *KRAS* gene amplification is grouped with *KRAS+*. The rounded values obtained for contours drawn by different operators using different type of segmentation algorithms within different software platforms are listed.

	Specificity	Sensitivity	Sensitivity + Specificity
**SUR_{MEAN}**			
**MIM**	**0.72**	**0.71**	**1.43**
**Hermes**	**0.81**	**0.64**	**1.46**
**SUR_{PEAK}**			
**MIM**	**0.66**	**0.75**	**1.40**
**Hermes**	**0.66**	**0.79**	**1.44**
**SUR_{MAX}**			
**MIM**	**0.72**	**0.75**	**1.47**
**Hermes**	**0.66**	**0.79**	**1.44**

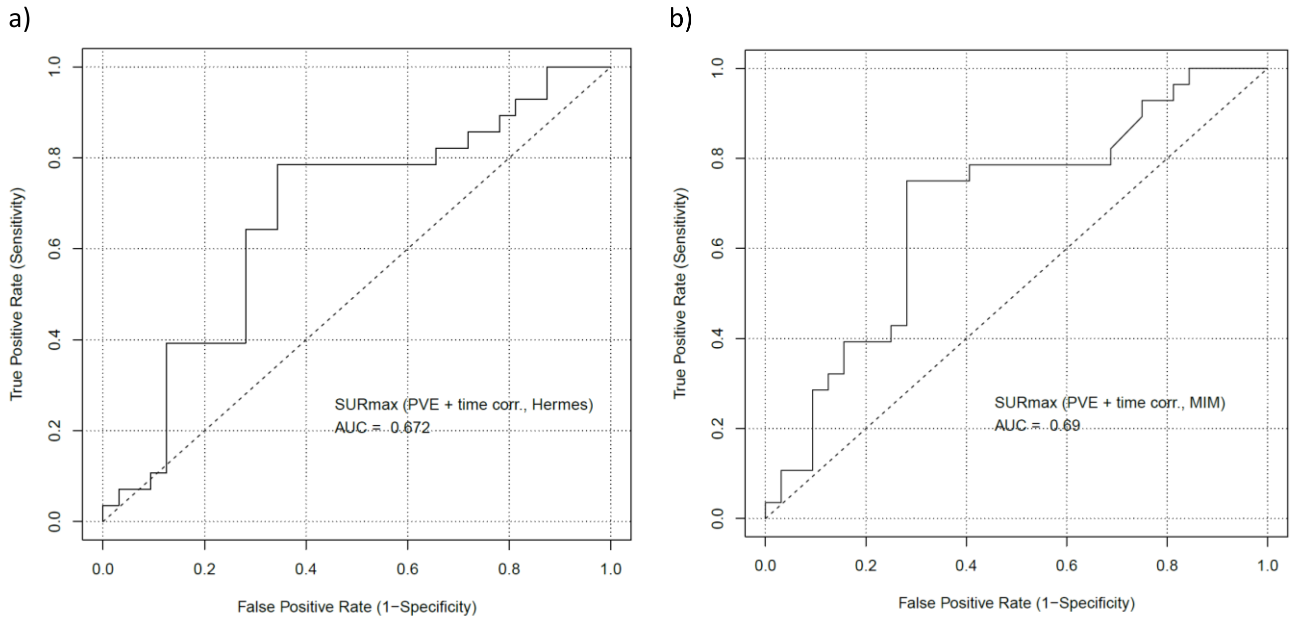

Fig. S1. Receiver operating characteristic (ROC) curves and AUC values for predicting CLM *KRAS* mutations (*KRAS* gene amplification is grouped with KRAS missense mutations) based on all 60 lesions and SUR_{MAX} with both corrections for Hermes (a, left) and MIM segmentations (b, right).

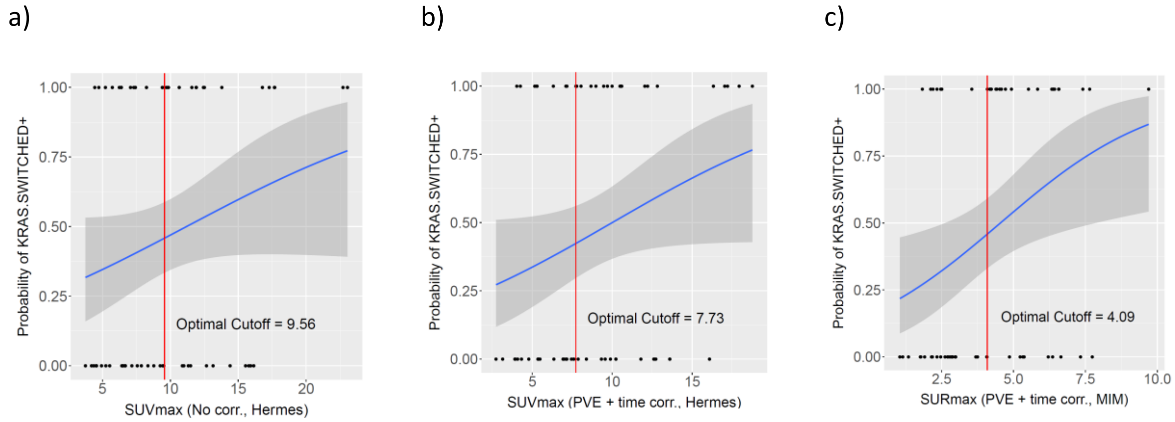

Fig. S2. Logistic regression curves based on all 60 lesions for SUV_{MAX} without any corrections (a), SUV_{MAX} with both PVE and uptake time corrections, (b), and for SUR_{MAX} with both corrections (c), when *KRAS* gene amplification is grouped with *KRAS* missense mutations. Dark gray shaded areas represent the 95% confidence intervals around the probability values. Individual data points are shown with dots at probability levels of 0.00 and 1.00.
